# Breech presentation and the cornual-fundal location of the placenta

**DOI:** 10.3325/cmj.2013.54.198

**Published:** 2013-04

**Authors:** Slobodan Sekulić, Marko Ilinčić, Gordana Radeka, Aleksandra Novakov-Mikić, Svetlana Simić, Jelena Podgorac, Goran Keković

**Affiliations:** 1 Department of Neurology, University Hospital, Clinical Center of Vojvodina, Novi Sad, Serbia; 2 Department of Obstetrics and Gynecology, University Hospital, Clinical Center of Vojvodina, Novi Sad, Serbia; 3 Institute of Biological Research “Dr Siniša Stanković,” Belgrade, Serbia

## Abstract

**Aim:**

To investigate the association of cornual-fundal location of the placenta and breech presentation at term delivery.

**Methods:**

This study was conducted at the Department of Obstetrics and Gynecology, Novi Sad, in 2011. The inclusion criteria were delivery at ≥37 weeks of gestation, singleton gestation, and cornual-fundal location of the placenta determined by ultrasonography at ≥37 weeks of gestation when 3/4 or more of the placenta was in the cornual-fundal region.

**Results:**

Out of 2750 ultrasound examinations performed, 143 showed cornual-fundal location of the placenta (frequency 5.2%). Eighty six cases had cephalic presentation (60.14%) and 57 (39.86%) had breech presentation. Of the remaining cases with non- cornual-fundal location, 2585 had cephalic presentation and 22 (0.84%) had breech presentation. The difference in the frequency of breech presentation between the cornual-fundal and non-cornual-fundal groups was significant (χ^2^ = 77.78, *P* < 0.001).

**Conclusion:**

Cornual-fundal location of the placenta may be an important clue in resolving the etiology of a number of cases of breech presentation at term delivery.

Breech presentation is defined as the position of the fetus with its buttocks or knees or feet closest to the cervix, and its head in the fundal region. Its frequency at birth is 3% (1-3). Breech presentation at birth increases the risk of deviation from normal delivery mechanisms and causes incomplete engaging of the presenting part of the fetus in the isthmic part of the uterus. This could be followed by a delay in delivery and an increased incidence of birth asphyxia because of umbilical cord prolapse and head entrapment (1). The role of the cornual-fundal location of the placenta in the etiology of breech presentation has not been fully resolved. Some studies found that it was the cause of breech presentation (2-4), but others did not establish this association (5). There is a lack of ultrasound data about the incidence of breech and cephalic presentation in cases of cornual-fundal location. The aim of the present study was to investigate the frequency of the cornual-fundal location of the placenta as well as the frequencies of breech and cephalic presentation in the cornual-fundal location of the placenta.

## Methods

This study was conducted at the Department of Obstetrics and Gynecology, Clinical Center of Vojvodina, Novi Sad, during 2011. Ultrasound examinations were performed in pregnant women admitted for delivery, using an apparatus Madison SonoAce ×8, convex probe 3.5 MHz (Samsung, Ridgefield Park, NJ, USA). The inclusion criteria were delivery at ≥37 weeks of gestation, singleton gestation, and the position of the placenta determined by ultrasonography at ≥37 weeks of gestation. Ultrasound examination at admission to hospital because of delivery is routinely performed at the Department of Obstetrics and Gynecology. However, the location of the placenta is classified only as anterior wall, posterior wall, fundal localization, placenta previa, and right and left wall. Cornual-fundal location is not routinely identified. Consequently, three authors of this article randomly repeated ultrasound examination and noted the cornual-fundal location of the placenta. The cases where it was visually estimated that 3/4 or more of the placenta was in the cornual-fundal region were classified as having the cornual-fundal location of the placenta. Ultrasound determination of the placental position is the gold standard, and an expected frequency of the cornual-fundal location of the placenta is between 5 and 7% (3,6,7). Since the frequency of breech presentation in the general population is significantly lower than in the cornual-fundal group (5% vs 35%, *P* < 0.01) and the desirable study power value of Type I error was 0.01 and Type II error was 0.01, it was estimated that the sufficient number of cases in the studied group should be more than 73. The planned number of examinations in this study was around 2500, and considering the stated frequency, we expected 125-170 cases with the cornual-fundal location of the placenta. The non cornual-fundal group included the first 200 cases with non-cornual-fundal location of the placenta, and the inclusion criteria were the same as in the cornual-fundal group. The following data were collected from the delivery room protocol: maternal age, number of pregnancies, parity, gestational age at delivery, sex, weight and length of the newborn, and mode of delivery (vaginal delivery or cesarean section). Consideration of a possible influence of external cephalic version on the results was not necessary, because this method was not used in the population covered by the study.

Kolmogorov-Smirnov test was used to test the normality of data distribution and the level of statistical significance was set at 5%. Considering that variables (number of pregnancies and deliveries, maternal age, weeks of gestation, newborn’s length and weight) were not normally distributed, non-parametric Mann-Whitney U test was used. χ^2^ test was used for comparison of differences in neonate’s sex and mode of delivery between the groups. The statistical tests were performed using the SPSS software (version 11.5.0, 2002, IBM, Armonk, NY, USA). Results of Mann-Whitney U test are presented as median and range and the results of χ^2^ square test as number and frequency.

## Results

The total number of births at the Department of Obstetrics and Gynecology during 2011 was 6327 and we collected the data on 2750. Out of these, 143 cases had cornual-fundal placental location (frequency 5.2%), 86 were in cephalic presentation at delivery (60.14%), and 57 were in breech presentation (39.86%). Among the cases with non-cornual-fundal location, 2585 were in cephalic presentation and 22 (0.84%) in breech presentation. The difference in the frequency of breech presentation between the cornual-fundal and the non-cornual-fundal groups was significant (χ^2^ = 77.78, *P* < 0.001). Cephalic presentation was in all cases associated with anterior occipital presentation at delivery. Cornual-fundal group had a significantly shorter body length and an increased incidence of cesarean section, which are the consequences of an increased proportion of breech presentation (Table 1, Table 2).

**Table 1:** General characteristics of the investigated groups

	Non-cornual-fundal placenta cases, median (range)	Cornual-fundal placenta cases, median (range)	*P* (Mann- Whitney U test)	Breech-presenting cases with cornual-fundal placenta, median (range)	*P* (Mann- Whitney U test)
No. of pregnancies	2 (1-11)	2 (1-8)	0.730	1 (1-6)	0.020*
No. of deliveries	2 (1-6)	1 (1-4)	0.900	1 (1-4)	0.015*
Gestation age	40 (37-41)	40 (37-42)	0.740	39 (37-41)	0.140
Body weight (g)	3530 (2529-5380)	3430 (2450-4790)	0.210	3430 (2650-4460)	0.450
Body length (cm)	50 (45-56)	50 (39-55)	0.019*	49 (46-55)	0.001*
Maternal age	29 (16-44)	29 (16-45)	0.350	30 (20-40)	0.840

*level of significance *P* < 0.05.

**Table 2:** Sex of the neonate and mode of delivery in the investigated groups

	Non-cornual-fundal placenta cases	Cornual-fundal placenta cases	*P* (χ^2^ test)	Breech-presenting cases with cornual-fundal placenta	*P* (χ^2^ test)
Sex of the neonate, n (%):					
female	102 (51)	51 (59.30)	0.245	33 (57.89)	0.442
male	98 (49)	35 (40.70)		24 (42.11)	
Mode of delivery, n (%):					
cesarean section	27 (13.5)	77 (53.84)	0.001	55 (96.49)	<0.001
vaginal delivery	173 (86.5)	66 (46.16)		2 (3.51)	

## Discussion

In our study, the frequency of the cornual-fundal location of the placenta was 5.2%, and 39.86% of these cases had breech presentation. The frequency of breech presentation in similar studies varied from 27.3% to 32.24% and the frequency of cephalic presentation varied from 67.76% to 72.7% (3,6,7). All studies found similar frequencies of breech presentation in spite of different diagnostic methods used for establishing the placental location (manual palpation of the placenta before delivery (6) or ultrasound (3,7, current study). The higher frequency of breech presentation at term birth in cases with cornual-fundal location of the placenta (27.3%-39.86%) than in general population (3%), along with the presence of the cornual-fundal location of the placenta in 44.68% to 72.6% of cases of breech presentation at term delivery (3,6,7), indicate that this location of the placenta may be an important clue in resolving the etiology of breech presentation.

Breech presentation at birth is more frequent among primiparas and is related to a higher rate of cesarean section than cephalic presentation (8). Also, newborns with breech presentation at delivery have worse physical characteristics than those with cephalic presentation (9). After breech presentation at delivery, women less frequently decide to have another pregnancy, which may explain the higher frequency of primiparas (8). The physical characteristics of newborns indicate suboptimal intrauterine development, and future studies should explore the link between the cornual-fundal location of the placenta and the suboptimal development.

The cornual-fundal location of the placenta is either a cause of or a condition associated with breech presentation, rather than its consequence, because the location of the placenta is determined at the very beginning of pregnancy, while the probability of breech presentation at term delivery occurs after the 24th gestation week (10). The fetus actively changes its intrauterine presentation using a whole range of movements such as kicking, twisting, and locomotion (11). The cornual-fundal location of the placenta can directly, mechanically, prevent the turning of the fetus from cephalic to breech presentation in two ways. The first possibility is that fetal presentation is conditioned by the correlation of the shapes of the fetus and the intrauterine cavity. This was referred to as the accommodation theory. The cornual-fundal location of the placenta is taken as a proof of this theory (2). In fetuses in flexed habitus, pelvic region together with legs creates a bigger pole than the head. When the placental location is outside the cornual-fundal region, the fundal section becomes the most spacious and the fetus positions itself in the cephalic presentation. If the placental location is in the cornual-fundal region, the isthmical region becomes more spacious and the fetus positions itself in the breech presentation (2,3). According to this hypothesis, it is expected that the majority of fetuses with the cornual-fundal location of the placenta will have the breech presentation. However, results of the present study indicate that this location is more often associated with the cephalic than breech presentation. Therefore, the accommodation theory could only partially explain the etiology of breech presentation.

The other way is by preventing spontaneous turning of the fetus. Hypothetically, this location may be associated with a decrease in the volume of intrauterine space. Up to 24 gestational weeks, the frequencies of breech and cephalic presentations are equal within the longitudinal situs. A defining characteristic of this period is that, before it ends, the fetus overgrows the intrauterine cavity. From the 25th to 36th week of gestation, there occurs an exclusive increase in the frequency of cephalic presentation with a proportional decrease in the frequency of breech presentation (12). If the fetus in this period does not have enough space, it will not turn from breech to cephalic presentation. Since before this period the probabilities of breech and cephalic presentations are equal, the frequency of breech presentation in case of the cornual-fundal location of the placenta should not exceed 50% (12,13). The distribution of breech presentation in different studies, including this study, was from 27.3% to 39.86%, which supports this hypothesis. It has been suggested that there is a high rate of unsuccessful external cephalic version in the case of cornual-fundal location of the placenta (14). Opposite to this opinion, other studies indicate that location of the placenta on the anterior wall presents unfavorable predictor for external cephalic version (15,16). These data, together with those presented in this study, suggest that a mechanical factor may play a role in preventing spontaneous fetal version.

The cornual-fundal location of the placenta may hypothetically, in an indirect way, affect the ability of the fetus to turn from breech to cephalic presentation. If this location of the placenta for some reason does not provide optimal nutrition for the fetus, then due to poor general condition, the fetus would not have enough strength for the spontaneous cephalic version.

Different authors used different study designs to study the relation between fetal presentation and placental location. Haruyama (17) did not observe the cornual-fundal location, but separately the cornual and the fundal locations. He found the cornual location in 59.57% and the fundal location in 14.89% of breech presentations, which in sum (74.46%) corresponds to other studies (3,6,7). Whitehead (18) reported the cornual-fundal location in 48.12% of breech presentations. The percentage of breech and cephalic presentations in the cornual-fundal group was 95.74% and 4.26%, respectively. These results were influenced by a biased study sample that included all breech presenting cases and cases of cephalic presentation only with low insertion of the placenta (18). In this way, cephalic-presenting cases with cornual-fundal location of the placenta were excluded. Witkop et al (19) found an increased frequency of fundal location of the placenta in non-vertex presentations at birth (breech presentation and transverse lie) compared with vertex presentation (9% vs 5%). Fell (20) and Stevenson (2) investigated the location of the placenta only in breech-presenting fetuses and found the frequency of cornual-fundal location in 22% and 100% of cases, respectively. Luterkort et al (5) did not find a difference in the frequency of the cornual-fundal location of the placenta between breech (15%) and cephalic presentation (19%) at birth, but they showed a distribution of breech ( ~ 36%) and cephalic ( ~ 64%) presentations in the cornual-fundal location of the placenta similar to our results. Their study (5) did not comprise all cases of breech and cephalic presentations at birth but only breech-presenting ones in the 33rd week of gestation. By the end of gestation, one half of these fetuses assumed cephalic presentation and served as controls. The high frequency of the cornual-fundal location of the placenta in term-birth fetuses that were still in breech presentation in the 33rd week of gestation indicates that there is still a possibility that this location of the placenta impedes spontaneous turning of the fetus. It is possible that random inclusion in the cephalic group may cause a decrease in the cornual-fundal location of the placenta in that group.

A limitation of the present study, similar to all previous studies, is that placenta locations were determined subjectively in relation to the reference points such as the anterior wall, posterior wall, fundus, etc. In term births, the placenta is attached to one fifth or more of the uterus (20) and is not always entirely located in just one region of the uterus. Future studies should attempt to standardize the method of localization of the placenta instead of the current practice based on subjective estimation.
